# Specialist pneumonia intervention nurse service improves pneumonia care and outcome

**DOI:** 10.1136/bmjresp-2020-000863

**Published:** 2021-08-04

**Authors:** Robert C Free, Matthew Richardson, Camilla Pillay, Kayleigh Hawkes, Julie Skeemer, Rebecca Broughton, Pranabashis Haldar, Gerrit Woltmann

**Affiliations:** 1 Department of Respiratory Sciences, University of Leicester, Leicester, UK; 2 Renal Unit, King's College Hospital, London, UK; 3 Respiratory Medicine Department, Glenfield Hospital, Leicester, UK; 4 Corporate Medical and Nursing, University Hospitals of Leicester NHS Trust, Leicester, UK

**Keywords:** pneumonia, respiratory infection

## Abstract

**Background:**

A specialist pneumonia intervention nursing (SPIN) service was set up across a single National Health Service Trust in an effort to improve clinical outcomes. A quality improvement evaluation was performed to assess the outcomes associated with implementing the service before (2011–2013) and after (2014–2016) service implementation.

**Results:**

The SPIN service reviewed 38% of community-acquired pneumonia (CAP) admissions in 2014–2016. 82% of these admissions received antibiotic treatment in <4 hours (68.5% in the national audit). Compared with the pre-SPIN period, there was a significant reduction in both 30-day (OR=0.77 (0.70–0.85), p<0.0001) and in-hospital (OR=0.66 (0.60–0.73), p<0.0001) mortality after service implementation, with a review by the service showing the largest independent 30-day mortality benefit (HR=0.60 (0.53–0.67), p<0.0001). There was no change in length of stay (median 6 days).

**Conclusion:**

Implementation of a SPIN service improved adherence to BTS guidelines and achieved significant reductions in CAP-associated mortality. This enhanced model of care is low cost, highly effective and readily adoptable in secondary care.

**Key messages**

- Implementing specialist nurse teams for CAP delivers improved guideline adherence and survival for patients admitted with the condition.
- This study demonstrates how a low-cost specialist pneumonia intervention nursing (SPIN) service focused on CAP sustainably inproves care and reduces crude and adjusted patient mortality.

## Introduction

Community-acquired pneumonia (CAP) is the leading cause of in-hospital mortality, with a crude rate of 5%–15%, rising to 30% for inpatients who are admitted to intensive care unit.^1^ Pneumonia is responsible for more hospital admissions and bed days than any other lung disease in the UK, and the annual healthcare costs to the National Health Service (NHS) associated with CAP are estimated to exceed £1 billion. Despite this, pneumonia has historically been a substantially underestimated, frequently neglected and underfunded condition in the UK. This was recognised in the recently published NHS Long Term Plan,^2^ which highlights CAP as an NHS research priority for new treatments and care pathways.

CAP mortality is known to be associated with disease severity and a number of scores have been developed to quantify this, of which CURB-65^3^ is implemented most widely in practice. These scores are intended to improve the provision of prompt and appropriate clinical care for pneumonia, and their benefit in guiding clinical intervention has been demonstrated in a national implementation project that reported lower 30-day in-hospital mortality associated with prompt radiological diagnosis and severity-based treatment.^4 5^ This suggests that prognosis in CAP is modifiable and can be improved with better models of healthcare delivery.

The University Hospitals Leicester NHS Trust (UHL) provides emergency admissions facilities for a catchment population of one million people. The Trust previously registered higher than expected externally reported summary hospital-level mortality indicators (SHMI)^6^ associated with primary diagnosis of CAP. In response, we created a dedicated specialist pneumonia intervention nursing (SPIN) service in 2013/2014 with the aim of (1) improving systematic CURB-65 scoring for CAP admissions; and (2) improving and accelerating adherence to key components of the British Thoracic Society pneumonia guidelines. This included radiological diagnosis and prompt antibiotic treatment within 4 hours of admission. In addition, the SPIN team led a Trust-wide educational and awareness-raising campaign on the importance of guidelines-based management of acute CAP cases and facilitated radiological follow-up after discharge.

In this 5-year retrospective cohort study, comparing periods before, during and after implementation of the SPIN service, we report on the effectiveness of this nurse-led programme of interventions in improving guideline adherence and the impact of the SPIN service on crude in-hospital mortality, SHMI and HSMR (hospital standardised mortality ratio).

## Methods

This manuscript follows the Standards for Quality Improvement Reporting Excellence 2.0 guidelines for study design and analysis.^7^

### The SPIN team

In 2013/2014 a new SPIN service was created, to which two nurses were appointed and tasked with implementation of key measures to improve diagnosis and prompt management of CAP admissions. This service improvement proposal had been fully supported by the NHS Trust Clinical Effectiveness team and was funded through the Commissioning for Quality and Innovation payments framework for 2 years before long-term funding was secured. No other systematic changes to care affecting this patient group were introduced during the same time period.

An assessment checklist based on the BTS pneumonia guidelines was devised (online supplemental file 1). This checklist was developed through engagement with ward nurses and junior doctors to maximise visibility and was printed on adhesive stickers for medical notes and in its final version served as an educational resource for awareness raising during the implementation period. Embedded management guidance was disseminated widely using wall posters in admission areas displaying A2 versions of the stickers, through the Trust communication team on the home page of the hospital intranet and as part of dedicated face-to-face education modules delivered by the SPIN team. The service was operational weekdays between 09:00 and 17:00. CAP admissions outside working hours and during weekends were reviewed the following working day.

10.1136/bmjresp-2020-000863.supp1 Supplementary data

**Figure 1:**
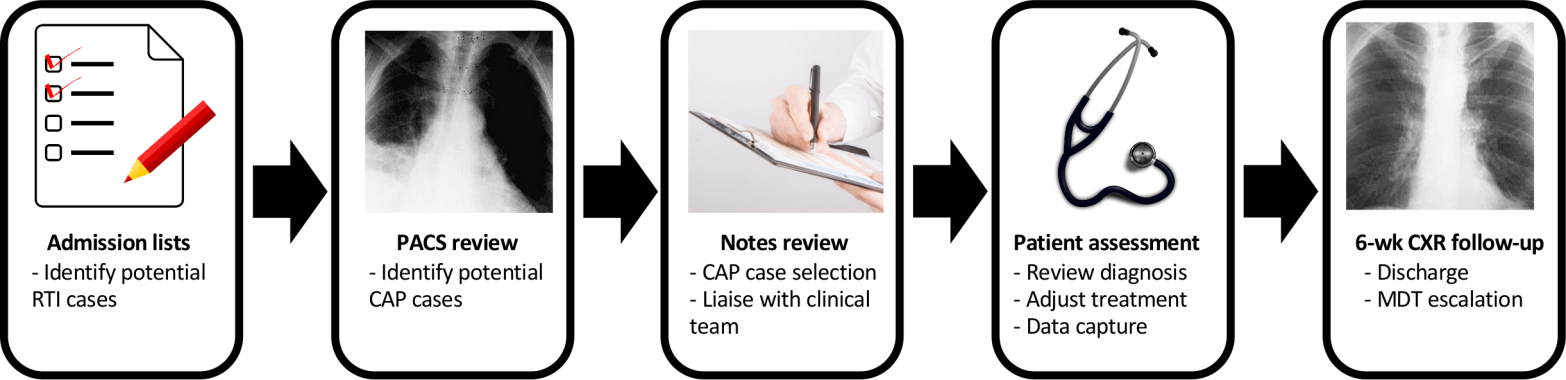
Schematic for admission screening of patients with CAP. Images used are in the public domain (available at https://commons.wikimedia.org and https://www.publicdomainpictures.net/en/view-image.php?image=164002). CAP, community-acquired pneumonia; CXR, chest X-ray; RTI, Respiratory Tract Infection; PACS, Picture Archiving and Communication System; MDT, Multi-disciplinary team.

Eligible emergency admissions were identified from daily ward list screening, review of admission chest X-rays on the picture archiving and communication system, and in response to electronic or verbal referral of patients with CAP (figure 1). Cases were predominantly seen in acute medical admission areas at both UHL emergency sites (Leicester Royal Infirmary and Glenfield Hospital). Severity scores (CURB-65) and interventions were prospectively recorded and time stamped for admissions physically seen by the SPIN team using bespoke intranet database facilities (National Institute for Health Research Biomedical Research Centre-Respiratory, Leicester).

### Data extraction and analysis

Data across five financial years (FY) were categorised into three periods for comparative analysis: (1) a 2-year baseline period (FY 2011/2012–2012/2013) prior to availability of the SPIN service; (2) a 1-year implementation phase (FY 2013/2014) during which the SPIN service was set up and operationalised; and (3) a 2-year intervention period (FY 2014/2015–2015/2016) when SPIN services were fully operational. This period was stratified further according to cases seen or not seen by the nursing service.

To ensure UHL admissions data were representative and unaffected by clinical coding bias, comparative per-hospital Trust admission frequency data were obtained from Telstra/Dr Foster Intelligence, a provider of healthcare information to stakeholders and the public in the UK, used to monitor the performance of the NHS.

Individual CAP coded hospital episodes from the study period were extracted from the hospital data warehouse by adapting the algorithm used by Telstra/Dr Foster for their quality and outcomes analysis. The International Classification of Diseases (ICD-10) clinical codes representing pneumonia in the Clinical Classifications Software^8^ group were used (online supplemental file 2) for this purpose. A patient and date/time matching algorithm linked CAP database intervention data to coded admission records, prior to anonymisation for analysis. Additional data fields derived from the anonymised admissions data were length of stay (LOS), Charlson Comorbidity Index (CCI)^6 9^ and time to death in days and weeks. CCI and age were categorised into groups of 0, 1–5 and >5 and into 16–65 and >65, respectively. The presence of heart disease and/or diabetes comorbidities on admission was determined by comparing secondary diagnoses with ICD-10 code lists (online supplemental file 3).

10.1136/bmjresp-2020-000863.supp2 Supplementary data

10.1136/bmjresp-2020-000863.supp3 Supplementary data

All statistical analyses were performed using R version 3.3.1.^10^ Comparisons between baseline and intervention variables were performed using the χ^2^ test, with Wald’s method used to calculate 95% CI. SHMI and HSMR adjusted outcome data were provided by Telstra/Dr Foster and included alongside the results.

Survival analysis used the Kaplan-Meir (KM) curves and Cox proportional hazards (PH) models. Patients were censored if they had died after the 1-year period examined or if they had survived beyond this time. KM survival analysis was carried out between baseline and intervention groups using time to death. A Cox PH model was constructed including potential confounding variables. Male gender and weekend admission were included as binary variables, and age and CCI as continuous variables.

### Patient and public involvement

The investigation did not include PPI in the design, analysis or interpretation of these data. However, extensive Trust-level engagement with managers and healthcare providers was undertaken to develop the resource.

## Results

### Descriptive CAP admission statistics

There were 13 496 CAP admissions to UHL between FY 2011 and 2016. The median age of admissions was 77.0 (IQR 64.0–86.0) years. Compared with the baseline period, there was an increase during the intervention period in the number of hospital admissions for CAP (4143 vs 7029). Standardised stationary series comparing monthly local CAP admission frequency at UHL with UK frequencies demonstrated concordance in the trend and seasonality of cases (online supplemental file 4). The rise in CAP admissions during the intervention period was accompanied by an increase in the proportion of patients with CAP aged >65 years (72.0% vs 73.8%, OR=1.09 (1.00–1.19), p<0.046) and the proportion with complex comorbidities (CCI >5) (from 41.9% to 47.8%, OR=1.27 (1.18–1.38), p<0.0001) (table 1).

10.1136/bmjresp-2020-000863.supp4 Supplementary data

**Table 1:** Comparison of admissions between baseline and SPIN intervention periods

	Baseline	SPIN intervention			
	% (n=4143)	% (n=7029)	OR	95% CI	P value
Male	52.1 (2159)	50.7 (3565)	0.95	0.88 to 1.02	0.155
Age					
16–65	28.0 (1158)	26.2 (1846)	0.92	0.84 to 1.00	**0.046**
>65	72.0 (2985)	73.8 (5187)	1.09	1.00 to 1.19	**0.046**
Charlson Comorbidity Index					
0	34.8 (1443)	28.8 (2024)	0.76	0.70 to 0.82	**<0.0001**
1–5	23.3 (966)	23.4 (1642)	1.00	0.92 to 1.10	0.957
>5	41.9 (1734)	47.8 (3363)	1.27	1.18 to 1.38	**<0.0001**
Weekend (Saturday/Sunday)	26.6 (1103)	27.8 (1953)	1.06	0.97 to 1.16	0.183

Significant results indicated in bold (p≤0.05)

SPIN, specialist pneumonia intervention nursing.

### Successful implementation and improved delivery of key interventions

Between implementation and the final interventional year, the SPIN nurses saw an increasing proportion of admitted CAP cases, rising from 34.1% (FY 2013/2014) to 42.0% (FY 2015/2016). Associated with this was a year-on-year improvement in compliance with guidelines-based interventions (table 2), with all interventions implemented in >80% of admissions seen by the service. In particular, the proportion of patients receiving antibiotics within 4 hours of admission (80.6%) and dual antibiotic treatment data for severe CAP (CURB-65 score 3–5) (86.3%) exceeded reported figures for these variables from the national audit^11^ (68.5% and 48.0%, respectively).

**Table 2:** CAP care bundle elements during implementation and intervention

Source	FY	Chest X-ray within 4 hours	Antibiotics given within 4 hours	CURB-65 recorded	Dual treatment for CURB-65 3–5 cases*
This paper	2013/2014	88.8 (703)	75.4 (596)	42.0 (333)	75.0 (36)
	2014/2015	89.1 (993)	83.3 (928)	72.3 (805)	87.6 (268)
	2015/2016	91.7 (1394)	80.6 (1227)	93.7 (1425)	86.3 (396)
Daniel *et al*^11^	2014	80.2	68.5	NA	48.0

*Dual treatment calculated for CURB 65 3–5 patients with drug information available (recording rate 89%).

CAP, community-acquired pneumonia; FY, financial years; NA, not available.

### Improved mortality

**Figure 2:**
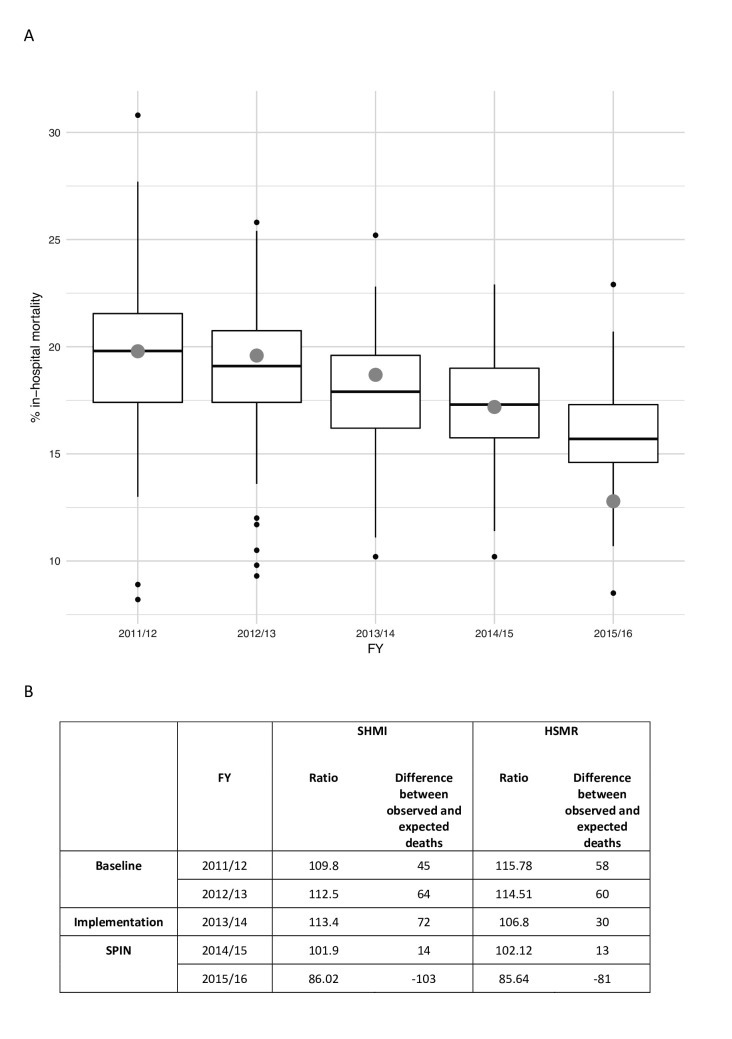
(A) Crude reported pneumonia-related mortality at UHL compared to other UK hospital trusts and (B) adjusted reported pneumonia-related mortality at UHL. FY, financial years; HSMR, hospital standardised mortality ratio; SHMI, summary hospital-level mortality indicators; SPIN, specialist pneumonia intervention nursing.

**Figure 3:**
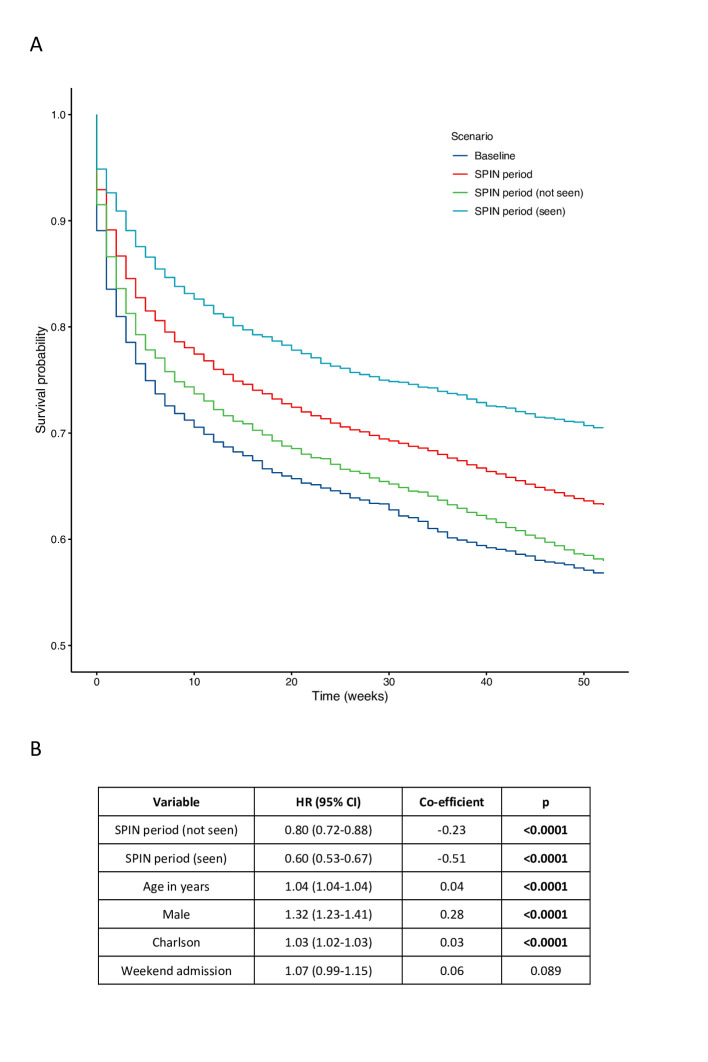
One-year survival analysis comparison for community-acquired pneumonia admissions for baseline (FY 2011/2012) and SPIN (FY 2015/2016) periods: (A) 1-year Kaplan-Meir survival curves for baseline and SPIN periods and (B) Cox proportional hazards model for 1-year survival. FY, financial years; SPIN, specialist pneumonia intervention nursing.

The overall crude in-hospital and 30-day mortality rates were 16.6% and 20.1%, respectively. This was aligned with hospital episode statistics (HES) derived crude mortality rates reported by all other NHS Trusts at baseline (figure 2). Externally reported adjusted mortality rates, SHMI and HSMR, improved year on year following the implementation period. Comparing the baseline period with the intervention period (table 3), a significant reduction in mortality was observed for both in-hospital mortality (OR=0.66 (0.60–0.73), p<0.0001) and 30-day mortality (OR=0.77 (0.70–0.85), p<0.0001), which was greatest in the subgroup of admissions seen by the SPIN nurses (in-hospital mortality: OR=0.49 (0.42–0.56), p<0.0001; 30-day mortality: OR=0.55 (0.48–0.63), p<0.0001). A smaller but significant reduction in mortality was also observed in the intervention period for patients not seen by the SPIN service (OR=0.77 (0.69–0.86), p<0.0001). The Cox PH model demonstrated being seen by the SPIN service to have had the largest independent effect on mortality reduction (HR=0.60 (0.53–0.67), p<0.0001) (figure 3).

**Table 3:** Outcomes for baseline compared with SPIN intervention period and whether patients were seen/not seen by service

	In-hospital				30-day (based on admission)			
	% (n)				% (n)			
Baseline	20.0 (829)				22.4 (928)			
		**OR**	**95%** **CI**	**P value**		**OR**	**95%** **CI**	**P value**
SPIN	14.2 (1000)	0.66	0.60 to 0.73	**<0.0001**	18.3 (1284)	0.77	0.70 to 0.85	**<0.0001**
Seen	10.9 (287)	0.49	0.42 to 0.56	**<0.0001**	13.7 (361)	0.55	0.48 to 0.63	**<0.0001**
Not seen	16.2 (713)	0.77	0.69 to 0.86	**<0.0001**	21.0 (923)	0.92	0.83 to 1.02	0.117

SPIN, specialist pneumonia intervention nursing.

The median LOS for CAP admissions (6 days) did not change significantly throughout the 5-year period and was unaffected by the SPIN service.

## Discussion

CAP remains the most common cause of in-hospital death and the UK ranks only 21st out of 99 countries for age-adjusted mortality.^12^ Although better adherence to the BTS pneumonia guidelines is recognised to be associated with improved survival trends in national audits,^5 11^ our experience suggests there is widely inconsistent implementation of this across different departments at large NHS organisations. The reasons for this are multifactorial and include widely fluctuating seasonal admission levels for respiratory tract infections, fluctuating medical staffing levels in and out of hours and inadequate provision of resource to support services for this common but neglected condition. Here we demonstrate how, for a large and geographically disparate NHS organisation, a novel SPIN service model, comprising only two specialist nurses working normal hours 5 days a week, could effectively overcome this variability in the provision of care. This model achieved sustained improvements in care bundle delivery that correlated with significant reductions in crude and adjusted mortality rates for hospital admissions with CAP. In particular, our observation that the SPIN intervention appeared to reduce 30-day mortality to 13.7%, compared with the reported UK average of 17.3%,^11^ supports the view that suboptimal care for CAP admissions is an important driver of national mortality rates and justifies prioritisation of CAP in the recently published NHS Long Term Plan.^2^

Implementation of the SPIN service improved outcomes in CAP cases that were not seen by the service, indicating the value and impact of education and awareness raising of CAP at a Trust-wide level. Given the high turnover in medical and nursing staff, sustaining this secondary benefit will require ongoing reinforcement of key messages by the service and should be viewed as an empirical component of the package of services that are provided.

There are some potential limitations to our analysis as we used a retrospective cohort design. However, it would be neither ethical nor feasible to perform a randomised prospective study to demonstrate effectiveness of these interventions for a condition known to have a poor outcome. Importantly our data confirm that rapid and robustly systematic implementation of BTS-mandated standards of care significantly improves CAP prognosis, independent of disease severity, age and existing comorbidities. The magnitude of the effect size we observed is at least equivalent to the development of new therapies for CAP and justifies the importance of improving care pathways for delivering existing resources.

Shortcomings of coded hospital episode data have been previously described, leading to concerns that CAP cases may be under-represented in HES data.^13 14^ However, our SPIN service model required the nurses to confirm the presence of consolidation on admission chest X-rays. We believe this provides a key checkpoint to increase the overall accuracy of pneumonia coding. To address the possibility that coding bias was introduced by adoption of the SPIN service, we compared our local hospital pneumonia coding frequencies with peer trusts and rates recorded across the UK using standardised stationary series and found our data to be concordant.

Selection bias may have been introduced when comparing outcomes for those seen or not seen by the SPIN team during the intervention period. Indeed, higher levels of comorbidities and increased mean age were evident in patients not reviewed by the SPIN team during the intervention period, which reflects a scarcity of resources within the existing SPIN service. However, a Cox PH model that included age and CCI as independent factors demonstrated the SPIN intervention to have the strongest independent effect on mortality (figure 3). On this basis, we intend to expand the service with recruitment of more specialist nurses, providing extended cover to facilitate more equitable access to the service for admitted patients with CAP.

The national pneumonia audit focused particularly on prescribing of dual antibiotics for cases of severe pneumonia (CURB-65 3–5). Our analysis examined this point in great detail and found significantly higher compliance with dual antibiotic prescribing compared with the national audit in patients seen by the SPIN team. UHL prescribing policy favours doxycycline over macrolides as the second antibiotic in severe pneumonia. This guidance remained consistent throughout the 5-year observation period.

Affecting more than 250 000 people in the UK annually, CAP continues to impact drastically on UK healthcare systems, and an ageing population is projected to lead to large increases in pneumonia admissions over the next two decades.^13^ In this context we conclude relatively simple specialist nurse-led interventions appear to provide an effective strategy with high impact at low cost. Wider adoption of the model across the acute care sector could help transform the outcome for large numbers of emergency admissions with this life-threatening condition.

## Data Availability

No data are available. This manuscript was the result of a service improvement exercise. Consequently, we do not have ethical approval to share the data used to produce the conclusions in this paper.

## References

[1] Chalmers JD, Campling J, Dicker A, et al. A systematic review of the burden of vaccine preventable pneumococcal disease in UK adults. BMC Pulm Med 2016;16:77. 10.1186/s12890-016-0242-0 27169895 PMC4864929

[2] NHS long term plan » the NHS long term plan. Available: https://www.longtermplan.nhs.uk/publication/nhs-long-term-plan/ [Accessed 15 Apr 2019].

[3] Lim WS, van der Eerden MM, Laing R, et al. Defining community acquired pneumonia severity on presentation to hospital: an international derivation and validation study. Thorax 2003;58:377–82. 10.1136/thorax.58.5.377 12728155 PMC1746657

[4] BTS guidelines for the management of community acquired pneumonia in adults: update 2009 | British thoracic Society | better lung health for all.. Available: https://www.brit-thoracic.org.uk/standards-of-care/guidelines/bts-guidelines-for-the-management-of-community-acquired-pneumonia-in-adults-update-2009/ [Accessed 26 Mar 2018].

[5] Lim WS, Rodrigo C, Turner AM, et al. British thoracic Society community-acquired pneumonia care bundle: results of a national implementation project. Thorax 2016;71:288–90. 10.1136/thoraxjnl-2015-206834 26197815

[6] Indicator specification: summary hospital-level mortality indicator (SHMI) 2018:38.

[7] Ogrinc G, Davies L, Goodman D, et al. Squire 2.0 (standards for quality improvement reporting excellence): revised publication guidelines from a detailed consensus process. BMJ Qual Saf 2016;25:986–92. 10.1136/bmjqs-2015-004411 PMC5256233 26369893

[8] Clinical classifications software (CCS) for ICD-10-CM/PCS. Available: https://www.hcup-us.ahrq.gov/toolssoftware/ccs10/ccs10.jsp [Accessed 28 Apr 2019].

[9] Charlson ME, Pompei P, Ales KL, et al. A new method of classifying prognostic comorbidity in longitudinal studies: development and validation. J Chronic Dis 1987;40:373–83. 10.1016/0021-9681(87)90171-8 3558716

[10] R Core Team. R: a language and environment for statistical computing. Vienna, Austria: R Foundation for Statistical Computing, 2013. http://www.R-project.org/

[11] Daniel P, Woodhead M, Welham S, et al. Mortality reduction in adult community-acquired pneumonia in the UK (2009-2014): results from the British thoracic Society audit programme. Thorax 2016;71:1061–3. 10.1136/thoraxjnl-2016-208937 27534681

[12] The battle for breath - the impact of lung disease in the UK. Br. Lung Found, 2016. Available: https://www.blf.org.uk/policy/the-battle-for-breath-2016

[13] Chalmers J, Campling J, Ellsbury G, et al. Community-Acquired pneumonia in the United Kingdom: a call to action. Pneumonia 2017;9:15. 10.1186/s41479-017-0039-9 29043150 PMC5628444

[14] Jarman B, Gault S, Alves B, et al. Explaining differences in English Hospital death rates using routinely collected data. BMJ 1999;318:1515–20. 10.1136/bmj.318.7197.1515 10356004 PMC27892

