# Supplementary material for: Specialist pneumonia intervention nurse service improves pneumonia care and outcome
Source: BMJ Open Respir Res. 2021 Aug 4;8(1):e000863. doi: 10.1136/bmjresp-2020-000863 (PMC8340276; doi:10.1136/bmjresp-2020-000863)
Supplement: Supplementary data [file bmjresp-2020-000863supp001.pdf]

Supplementary File 1: CAP checklist

Patient details sticker	4hr Community Acquired Pneumonia Care Bundle<br>Date of admission:<br>Time of admission:

**B**reathlessness, cough and pyrexia	Symptoms suggestive of LRTI	Yes ☐<br>No ☐
**R**adiology reviewed	New consolidation	Yes ☐<br>No ☐
**E**WS	Use EWS escalation tool<br>Fluid if SBP < 90<br>Consider catheter	EWS Score ☐
**A**ssess Severity CURB65	**C**onfusion<br>**U**rea >7<br>**R**R > 30<br>**B**P – SBP < 90 DBP <60<br>**65** age > 65<br>**Total**	☐<br>☐<br>☐<br>☐<br>☐<br>☐
**T**reatment	Use UHL guidance<br>(see diagram below)	☐
**H**ypoxia treated in 1st hour	Target sats. ≥94%<br>COPD 88-92%	☐<br>☐
**E**xpert advice	Bleep pneumonia nurse (2713)<br>Virtual respiratory clinic<br>(mobile / ICM)	☐<br>☐

Score 0-1 ☐	Score 2 ☐	Score 3-5 ☐
Amoxycillin 500 mg po tds 5/7<br>or<br>**if Penicillin allergic:**<br>Doxycycline 200 mg po od 5/7	Amoxycillin 1 g po tds 5/7<br>Doxycycline 200 mg po od 5/7<br>or<br>**if Penicillin allergic:**<br>Doxycycline 200 mg po od 5/7	Coamoxiclav 1.2g tds IV 5/7<br>Doxycycline 200 mg po od 5/7<br>or<br>**if Penicillin allergic:**<br>Doxycycline 200 mg po od 5/7<br>IV meropenem 500mg tds 5/7<br>**If anaphylaxis to penicillin discuss with microbiology**

Time Antibiotics given: (= bundle completion)	Name of Doctor:	Grade of Doctor:
