# Supplementary material for: Specialist pneumonia intervention nurse service improves pneumonia care and outcome
Source: BMJ Open Respir Res. 2021 Aug 4;8(1):e000863. doi: 10.1136/bmjresp-2020-000863 (PMC8340276; doi:10.1136/bmjresp-2020-000863)
Supplement: Supplementary data [file bmjresp-2020-000863supp002.pdf]

Supplementary File 2: ICD10 codes used to extract CAP episodes

A01.0, A02.2, A20.2, A21.2, A22.1, A31.0, A37.0, A37.1, A43.0, A48.1, B01.2, B05.2, B37.1, B38.0, B38.1, B38.2, B39.0, B39.1, B39.2, B58.3, B59, B77.8, J12.0, J12.1, J12.2, J12.3, J12.8, J12.8, J12.9, J13, J14, J15.0, J15.1, J15.2, J15.3, J15.4, J15.5, J15.6, J15.7, J15.8, J15.9, J16.0, J16.8, J17.0, J17.1, J17.2, J17.3, J17.8, J18.0, J18.1, J18.8, J18.9, J85.1, J85.2, B59.
