# Supplementary material for: Specialist pneumonia intervention nurse service improves pneumonia care and outcome
Source: BMJ Open Respir Res. 2021 Aug 4;8(1):e000863. doi: 10.1136/bmjresp-2020-000863 (PMC8340276; doi:10.1136/bmjresp-2020-000863)
Supplement: Supplementary data [file bmjresp-2020-000863supp003.pdf]

Supplementary File 3: ICD10 codes used to determine co-morbidities in CAP admissions

Heart disease:

I05.0, I05.1, I05.2, I05.8, I05.9, I06.0, I06.1, I06.2, I06.8, I06.9, I07.0, I07.1, I07.2, I07.8, I07.9, I08.0, I08.1, I08.2, I08.3, I08.8, I08.9, I09.1, I09.8, I34.0, I34.1, I34.2, I34.8, I34.9, I35.0, I35.1, I35.2, I35.8, I35.9, I36.0, I36.1, I36.2, I36.8, I36.9, I37.0, I37.1, I37.2, I37.8, I37.9, R00.8, R00.9, R01.0, R01.1, R01.2, Z95.2, Z95.3, Z95.4, A36.8, A38.1, A39.5, B26.8, B33.2, B37.6, B58.8, I01.0, I01.1, I01.2, I01.8, I01.9, I02.0, I09.0, I09.2, I09.9, I23.0, I30.0, I30.1, I30.8, I30.9, I31.0, I31.1, I31.2, I31.3, I31.4, I31.8, I31.9, I32, I33.0, I33.9, I38, I39, I40.0, I40.1, I40.8, I40.9, I41, I42.0, I42.1, I42.2, I42.3, I42.4, I42.5, I42.7, I42.8, I42.9, I43, I51.4, J10.8, I10, I11.0, I11.9, I12.0, I12.9, I13.0, I13.1, I13.2, I15.0, I15.1, I15.2, I15.8, I15.9, I67.4, N26.2, I21.0, I21.1, I21.2, I21.3, I21.4, I22.0, I22.1, I22.2, I22.8, I22.9, I20.0, I20.1, I20.8, I20.9, I23.7, I24.0, I24.1, I24.8, I24.9, I25.1, I25.2, I25.5, I25.6, I25.7, I25.8, I25.9, Z95.1, Z95.5, Z98.6, I26.0, I26.9, I27.0, I27.1, I27.2, I27.8, I27.9, I28.0, I28.1, I28.8, I28.9, Z86.7, I23.1, I23.2, I23.3, I23.4, I23.5, I23.6, I23.8, I25.3, I25.4, I51.0, I51.1, I51.2, I51.3, I51.5, I51.7, I51.8, I51.9, I52, I47.0, I47.1, I47.2, I47.9, I48.0, I48.1, I48.2, I48.3, I48.4, I48.9, I49.1, I49.2, I49.3, I49.4, I49.5, I49.8, I49.9, R00.0, R00.1, R00.2, I46.2, I46.8, I46.9, I49.0, I50.1, I50.2, I50.3, I50.4, I50.9, P29.3, Q20.0, Q20.1, Q20.2, Q20.3, Q20.4, Q20.5, Q20.6, Q20.8, Q20.9, Q21.0, Q21.1, Q21.2, Q21.3, Q21.4, Q21.8, Q21.9, Q22.0, Q22.1, Q22.2, Q22.3, Q22.4, Q22.5, Q22.6, Q22.8, Q22.9, Q23.0, Q23.1, Q23.2, Q23.3, Q23.4, Q23.8, Q23.9, Q24.0, Q24.1, Q24.2 Q24.3, Q24.4, Q24.5, Q24.6, Q24.8, Q24.9, Q25.0, Q25.1, Q25.2, Q25.3, Q25.4, Q25.5, Q25.6, Q25.7, Q25.8, Q25.9, Q26.0, Q26.1, Q26.2, Q26.3, Q26.4, Q26.5, Q26.6, Q26.8, Q26.9, Q27.0, Q27.1, Q27.2, Q27.3, Q27.4, Q27.8, Q27.9, Q28.0, Q28.1, Q28.2, Q28.3, Q28.8, Q28.9, Z87.7

Diabetes:

E08.9, E09.9, E10.9, E11.9, E13.9, R73.0, R73.9, R81, R82.4, Z46.8, Z96.4, E08.0, E08.1, E08.2, E08.3, E08.4, E08.5, E08.6, E08.8, E09.0, E09.1, E09.2, E09.3, E09.4, E09.5, E09.6, E09.8, E10.1, E10.2, E10.3, E10.4, E10.5, E10.6, E10.8, E11.0, E11.2, E11.3, E11.4, E11.5, E11.6, E11.8, E13.0, E13.1, E13.2, E13.3, E13.4, E13.5, E13.6, E13.8, G32.8, K85.0, K85.1, K85.2, K85.3, K85.8, K85.9, K86.0, K86.1, K86.2, K86.3, K86.8, K86.9, K90.3, O24.0, O24.1, O24.3, O24.4, O24.8, O24.9, O99.8, Z86.3

Codes used are based on Clinical Classification System (https://www.hcup-us.ahrq.gov/toolssoftware/ccs10/ccs10.jsp) code group categories extracted using regular expressions:
“Cardiac|cardiac|Heart|heart|hypertension|hypotension|infarction|cardiomyopathy” and “diabetes|Diabetes“
