# Supplementary material for: Specialist pneumonia intervention nurse service improves pneumonia care and outcome
Source: BMJ Open Respir Res. 2021 Aug 4;8(1):e000863. doi: 10.1136/bmjresp-2020-000863 (PMC8340276; doi:10.1136/bmjresp-2020-000863)
Supplement: Supplementary data [file bmjresp-2020-000863supp004.pdf]

Supplementary File 4: Differences between standardised stationary series for UHL CAP admissions and national CAP admissions

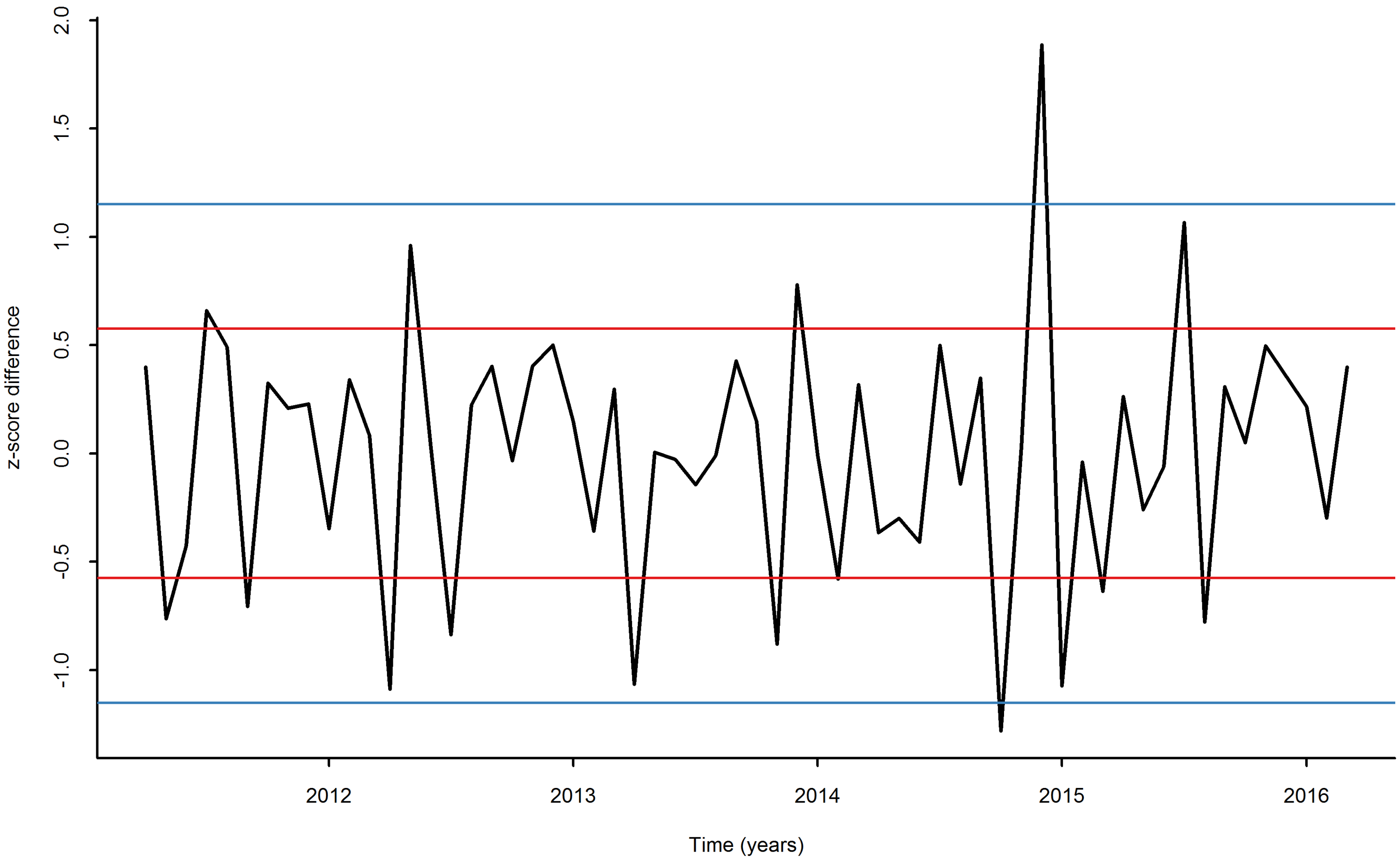

Aggregate monthly admission data was kindly supplied by Dr Foster.
Blue and red lines indicate 1 and 2 standard deviations above the mean difference respectively.
